# Two-Pore Channels: Catalyzers of Endolysosomal Transport and Function

**DOI:** 10.3389/fphar.2017.00045

**Published:** 2017-02-07

**Authors:** Christian Grimm, Cheng-Chang Chen, Christian Wahl-Schott, Martin Biel

**Affiliations:** ^1^Center for Integrated Protein Science Munich, Ludwig Maximilian University of MunichMunich, Germany; ^2^Department of Pharmacy – Center for Drug Research, Ludwig Maximilian University of MunichMunich, Germany

**Keywords:** calcium, TPC, two-pore, lysosome, TPC1, TPC2

## Abstract

Two-pore channels (TPCs) have recently emerged as a novel class of non-selective cation channels in the endolysosomal system. There are two members in the human genome, TPC1 and TPC2. Studies with TPC knockout and knockdown models have revealed that these channels participate in the regulation of multiple endolysosomal trafficking pathways which when dysregulated can lead to or influence the development of a range of different diseases such as lysosomal storage, metabolic, or infectious diseases. TPCs have been demonstrated to be activated by different endogenous stimuli, PI(3,5)P_2_ and NAADP, and ATP has been found to block TPC activation via mTOR. Loss of TPCs can lead to obesity and hypercholesterolemia, and to a slow-down of intracellular virus and bacterial toxin trafficking, it can affect VEGF-induced neoangiogenesis, autophagy, human hair pigmentation or the acrosome reaction in sperm. Moreover, physiological roles of TPCs in cardiac myocytes and pancreatic β cells have been postulated.

## Introduction

Since its development by Neher and Sakmann (1976), the patch clamp technique has been successfully applied to study ion channels in the plasma membrane. In the 1980s and 1990s ion channels in inner and outer membranes of mitochondria (Sorgato et al., 1987), ion channels in chloroplast envelope membranes (Pottosin et al., 1993; Heiber et al., 1995), and in thylakoid membranes (Pottosin and Schönknecht, 1995) have been patch clamped successfully. Just recently, it became possible to also patch-clamp ion channels in endolysosomal membranes. Such endolysosomal ion channels comprise mucolipins (TRPML channels; Dong et al., 2010; Shen et al., 2012; Samie et al., 2013; Cheng et al., 2014; Sakurai et al., 2015; Li et al., 2016), P2X4 (Qureshi et al., 2007; Huang et al., 2014; Zhong et al., 2016), TMEM175 (Cang et al., 2015), BK (Cao et al., 2015), CLCs (Jentsch et al., 2015; Zifarelli, 2015), TRPM2 (Lange et al., 2009), and Two-pore channels (TPCs; Wang et al., 2012; Cang et al., 2013, 2014; Grimm et al., 2014). With this novel, modified patch clamp technique endolysosomal ion channels can be studied directly in their native membranes. This has undoubtedly contributed to a better understanding of the function and physiology of endolysosomal ion channels such as the TPCs. TPCs derive their name from the two pore domains that are found on each subunit. TPC subunits consist of 12 transmembrane domains (TMDs) with the putative pore loops between TMD5/6 and TMD11/12, respectively. Two subunits dimerize to form one functional pore. With this topology, the TPCs are residing between the six TMD containing cation channels such as, e.g., the TRP channels including the endolysosomal TRPML channels and the voltage-gated cation channels (Ca_v_s and Na_v_s) with their 24 TMDs. Humans have two isoforms, TPC1 and TPC2. Using a combination of knockout mouse and knockdown models, cell biological methods, imaging technologies and the endolysosomal patch clamp technique, the functions of these channels have been explored in more detail in recent years.

## Roles in Endolysosomal Function

### TPCs: Mediators of Endolysosomal Vesicle Fusion?

Two-pore channels have been found to be involved in the trafficking of viruses such as Ebola virus (EBOV; Sakurai et al., 2015) as well as bacterial toxins such as cholera toxin (CT; Ruas et al., 2010, 2014). Furthermore, in TPC2^-/-^ cells EGF/EGFR, PDGFR, and LDL-cholesterol trafficking were found to be affected (Grimm et al., 2014; Ruas et al., 2014).

In independent interactome screens novel interaction partners of TPCs have been identified (Grimm et al., 2014; Lin-Moshier et al., 2014). The results published by Grimm et al. (2014) and Lin-Moshier et al. (2014) have nicely been summarized in a recent review by Marchant and Patel (2015). We employed the quantitative proteomic method of SILAC (stable isotope labeling by amino acids in cell culture)-immunoprecipitation to identify TPC2 interacting proteins while Lin-Moshier et al. (2014) used an affinity purification method for protein complexes based upon ‘one-Strep’-tagging. Many interaction candidates were found in both independent interactome screens and a considerable number of those proteins are proteins involved in the regulation of intracellular vesicle trafficking and fusion processes, e.g., syntaxins (STX), VAMP and VTI proteins, or Rabs. Marchant and Patel (2015) concluded in their review: “Overall, only four out of 26 proteins identified by Grimm et al. (2014) are not represented in the study by Lin-Moshier et al. (2014), despite the different proteomic procedures and methods employed in the two studies (difference in tags, tag orientation, TPC isoforms used). This convergence brings considerable confidence to the association between TPCs and these trafficking regulators […].”

**FIGURE 1:**
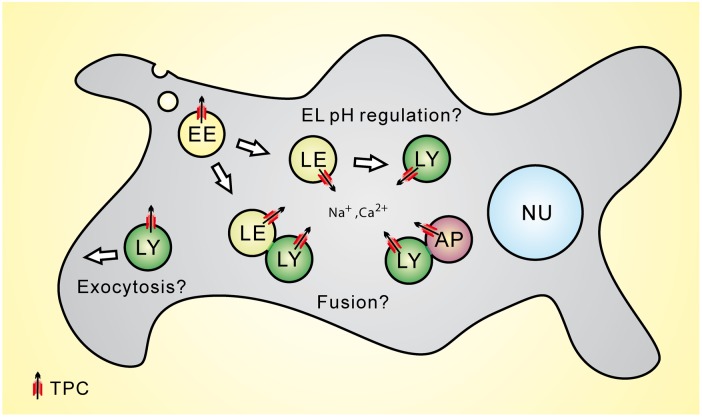
**Subcellular location of Two-pore channels (TPCs) and potential functional roles of TPCs within the endolysosomal system**. LE, late endosome; LY, lysosome; AP, autophagosome.

The putative interactions between TPC2 and STX7 as well as STX6 were further confirmed by FRET analyses and co-immunoprecipitation experiments (Grimm et al., 2014) while the interaction between Rab7 and TPC2 was corroborated by co-immunoprecipitation experiments and functional assays (Lin-Moshier et al., 2014). STX such as STX7 and STX6 are forming complexes with other SNARE proteins to control fusion processes between different intracellular vesicles and organelles. For example, fusion between late endosomes and lysosomes requires the interaction between STX7, STX8, VTI1b, and VAMP7 (Prekeris et al., 1999; Antonin et al., 2000; Mullock et al., 2000; Luzio et al., 2009, 2010) while trafficking from early endosomes to the TGN (*trans*-Golgi network) requires STX6, STX16, VTI1a, and VAMP4 (Jung et al., 2012). STX6 further regulates endocytic recycling and chemotactic cell migration (Riggs et al., 2012) and may be involved in other endolysosomal trafficking processes (Jung et al., 2012). Importantly, there is evidence from cell-free content mixing assays that Ca^2+^ released from the lumen of the fusing organelles is required for both homotypic and heterotypic fusion events in the endocytic pathway (Luzio et al., 2007). The molecular identity of the Ca^2+^ release channel remains to be elucidated. Whether TPCs may be a sufficient Ca^2+^ source providing this signal needs to be further established (**Figure 1**).

### TPCs: Regulators of Organellar pH?

Two-pore channels have also been postulated to play a role in the control of the luminal pH of endolysosomal vesicles (**Figure 1**). Lin et al. (2015) reported that TPC2^-/-^ myoblast lysosomes exhibit a more alkaline lysosomal pH under resting conditions and Lu et al. (2013a) proposed that TPC2 overexpression in HeLa cells results in a more alkaline lysosomal pH. However, in a number of studies, lysosomal pH was found not to be affected in TPC2^-/-^ cells under resting conditions, e.g., in knockout fibroblasts or macrophages (Cang et al., 2013; Grimm et al., 2014; Ruas et al., 2014). Cang et al. (2013) proposed that under both starvation and non-starvation conditions WT macrophage lysosomes maintain a stable pH while TPC2^-/-^ macrophage lysosomes showed a significant shift toward alkalization after starvation. Recently, Ambrosio et al. (2016) as well as Bellono et al. (2016) reported a role for TPC2 in melanosomes, the melanin producing organelles of melanocytes. They found that loss of TPC2 leads to an increase in melanosomal pH and an increase in melanin production while overexpression of TPC2 leads to a decrease in melanin production. As the optimal enzymatic activity of the key enzyme of melanin production, tyrosinase, is above pH 6, melanosomal acidification by TPC2 activation or overexpression would likely decrease its activity and thus the melanin biosynthesis (Patwardhan and Delevoye, 2016). Those studies on the role for TPC2 in melanosomes were preceded by a publication by Sulem et al. (2008) who presented results from a genome-wide association study for variants associated with human pigmentation characteristics among 5,130 Icelanders, with follow-up analyses in 2,116 Icelanders and 1,214 Dutch individuals from which they had claimed that two coding variants of hTPC2 (SNPs), namely M484L and G734E, were associated with a shift from brown to blond hair color. How exactly these polymorphisms impact the human TPC2 channel characteristics and functions remains, however, to be elucidated. Likewise, it needs to be further established under which conditions TPC2 activity affects endolysosomal and lysosome-related organelle pH and whether there are differences between different cell types.

### TPCs: Direct Targets of mTOR

Cang et al. (2013) demonstrated direct interaction between TPCs and mTOR (mammalian or mechanistic target of rapamycin) while the endolysosomal TRPML cation channels did not interact with mTOR. mTOR integrates the input from upstream pathways, including growth factors (such as IGF-1 and IGF-2), insulin, and amino acids. It also senses cellular nutrient, oxygen, and energy levels and thus serves as a central regulator of mammalian metabolism and physiology, with important roles for liver, muscle, white and brown adipose tissue, and brain function. In human diseases such as diabetes, obesity, and cancer mTOR is dysregulated. TPCs detect nutrient status and become constitutively active upon nutrient removal (Cang et al., 2013). Whether this exciting crosstalk between mTOR and TPCs may be exploited therapeutically remains to be seen.

## Activation Mechanisms

In 2009 several groups provided evidence that TPCs are activated by NAADP (nicotinic acid adenine dinucleotide phosphate), a Ca^2+^-mobilizing second messenger which had been discovered in Clapper et al. (1987) together with cADPR (cyclic ADP ribose; Brailoiu et al., 2009; Calcraft et al., 2009; Zong et al., 2009). This finding, that TPCs are NAADP-activated calcium release channels on endolysosomal membranes was challenged by Wang et al. (2012) as well as Cang et al. (2013) who claimed that TPCs are Na^+^ selective channels activated by PI(3,5)P_2_ (phosphatidylinositol 3,5-bisphosphate) and not by NAADP. Although there is a consensus that TPCs can, under various conditions and recording configurations, be activated by PI(3,5)P_2_, it is unclear up to now why NAADP activation of TPCs is less consistently observed. A number of recent reviews, e.g., by Jentsch et al. (2015), Marchant and Patel (2015), or Ruas et al. (2015b) have summarized the current debate in detail. Nevertheless, a few critical points shall be repeated here. First, Jha et al. (2014) found that in the absence of Mg^2+^, NAADP-evoked TPC2-mediated Na^+^ currents are readily detectable using the same recording configuration as Wang et al. (2012). This suggests that Mg^2+^ may have impacted the detection of NAADP currents in the previous studies. Secondly, NAADP activation of TPCs may require an auxiliary protein (Lin-Moshier et al., 2012; Walseth et al., 2012; Morgan et al., 2015; Ruas et al., 2015b) which may be missing or may be diluted under certain experimental conditions. Finally, concern about the validity of TPC knockout mouse models used to show that NAADP-responses are still present in the absence of TPCs was raised (Ruas et al., 2015b). Recently, Ruas et al. (2015a) generated a novel TPC-null mouse line with demonstrable absence of both TPC1 and TPC2. In this study Ruas et al. (2015a) show that the loss of endogenous TPCs abolished NAADP-dependent Ca^2+^ responses as assessed by single-cell Ca^2+^ imaging or endolysosomal patch-clamp. NAADP-sensitivity was restored by re-expressing wild-type TPCs, but not by mutant versions with impaired Ca^2+^-permeability. Ruas et al. (2015a) further reported that the mouse line formerly described as TPC-null (Wang et al., 2012; Cang et al., 2013) expresses truncated TPCs but that these truncated TPCs still support NAADP-induced Ca^2+^ release.

Nevertheless, it will be necessary in the future to determine in more detail under which conditions NAADP responses are detectable and under which conditions they are not, what influence technical characteristics (e.g., the size of measured endolysosomes) may have and what the identity of the putative intermediator protein may be. In addition, it remains to be clarified what the upstream signals are that lead to the formation of either NAADP or PI(3,5)P_2_ prior to TPC activation.

One fundamental difference between TPC1 and TPC2 is their voltage dependence. Cang et al. (2014) reported that TPC1 is a voltage-gated, non-inactivating Na^+^ channel, which they called lysoNaV and which is formed only by TPC1 but not by TPC2. Luminal alkalization was found to also open TPC1 by shifting the channel’s voltage dependence of activation toward hyperpolarization. The authors concluded that endolysosomes sense both voltage and pH changes with lysoNaV (Cang et al., 2014).

Interestingly, in other studies it was found that luminal alkalization inhibits NAADP evoked TPC2 Ca^2+^ currents while PI(3,5)P_2_ evoked TPC2 Na^+^ currents are not inhibited (Schieder et al., 2010; Wang et al., 2012; Jha et al., 2014). Taken together these findings suggest that TPC1 and TPC2, despite significant similarities in sequence and function may differ substantially in their overall activation mechanisms.

## TPCs and Disease Relevance

**FIGURE 2:**
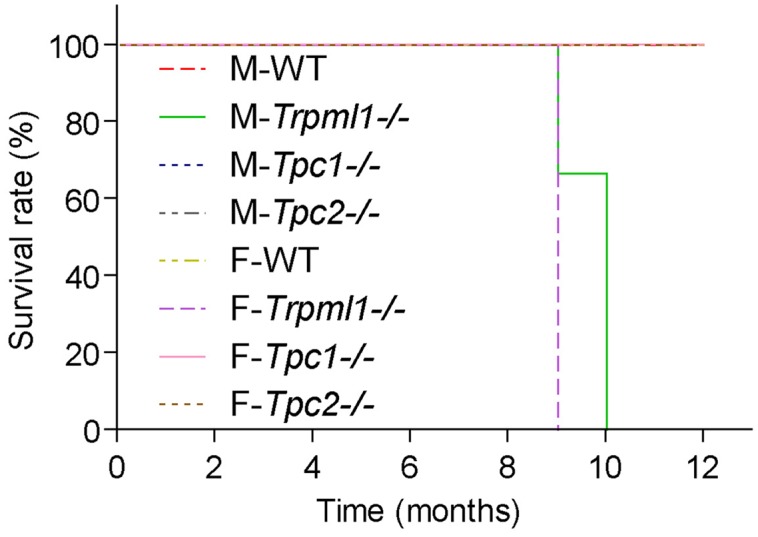
**Kaplan–Meier plots depicting the survival rates of male and female TRPML1^-/-^, TPC1^-/-^, TPC2^-/-^, and WT mice (*n* = 5-10 mice, each)**. Mice were fed a standard diet and kept under normal husbandry conditions. TRPML1^-/-^ mice typically died or had to be euthanized due to severe paralysis between 9 and 10 month of age.

Studies with TPC knockout and knockdown models have revealed that TPCs participate in the regulation of multiple endolysosomal trafficking pathways which when dysregulated can lead to or influence the development of a range of different diseases such as lysosomal storage, metabolic, infectious diseases, or cancer. Thus, it was found that loss of TPCs can lead to or accelerate the development of hypercholesterolemia and fatty liver hepatitis under certain dietary conditions (Grimm et al., 2014), can slow-down intracellular virus and bacterial toxin trafficking (Ruas et al., 2010, 2014; Sakurai et al., 2015), can affect VEGF-induced neoangiogenesis (Favia et al., 2014), autophagy (Lu et al., 2013a,b; Fernández et al., 2016), and human hair pigmentation (Sulem et al., 2008; Ambrosio et al., 2016; Bellono et al., 2016), and the acrosome reaction in sperm (Arndt et al., 2014). Moreover, physiological roles of TPCs in cardiomyocytes (Capel et al., 2015; Davidson et al., 2015) and pancreatic β cells (Arredouani et al., 2015) have been postulated. Most of these proposed physiological roles and functions have in common that endolysosomal trafficking processes and/or endolysosomal cation homeostasis are disrupted or disturbed to a certain degree. Importantly, however, TPC1 as well as TPC2 knockout mice have no reduced life span or an obvious reduction in quality of life in contrast to mice in which the endolysosomal cation channel TRPML1 is knocked out (Grishchuk et al., 2014; **Figure 2**). This suggests that in contrast to TRPML1, the system is able to somehow compensate for the loss of one or both TPCs. Hence, TPCs may be infact more relevant under stress and under challenging environmental conditions than under basal conditions.

## Conclusion

A role for TPCs in promoting endolysosomal trafficking appears to be well supported by the literature. Its activation in endolysosomal vesicles by PI(3,5)P_2_ or NAADP has been confirmed by several research groups, although there is still an ongoing debate on the exact conditions under which TPCs are activated by NAADP and whether a mediator protein may be required for this activation. The identity of such a mediator needs to be determined. Interaction with SNARE proteins has been found by at least two independent groups, likewise data on the interaction with mTOR is very convincing. Unclear is the role in endolysosomal pH regulation, the role of TPCs in autophagy needs to be further elucidated and whether TPCs play a role in endolysosomal vesicle fusion of fission is lacking final proof. Whether PI(3,5)P_2_ or NAADP are endogenous TPC ligands also remains uncertain. That TPCs are disease relevant has, however, been convincingly demonstrated by several studies. The fact that TPCs have been found to be relevant for a multitude of diseases will certainly encourage further research in this field. Both activation and inhibition of TPCs may be beneficial depending on the disease or condition to be treated, e.g., TPC inhibition may be beneficial in infectious diseases to prevent effective transport of virus (e.g., Ebola) or bacterial toxins (e.g., CT) in the endolysosomal system. On the other hand, TPC activation may promote, e.g., LDL cholesterol trafficking and may thus prevent subcellular accumulation of too much cholesterol. Likewise the accumulation of macromolecules and other material in lysosomes of neurons may be reverted or prevented by activating TPCs on time.

## Author Contributions

All authors listed, have made substantial, direct and intellectual contribution to the work, and approved it for publication.

## Conflict of Interest Statement

The authors declare that the research was conducted in the absence of any commercial or financial relationships that could be construed as a potential conflict of interest.
